# Analysis of recycled poly (styrene-co-butadiene) sulfonation: a new approach in solid catalysts for biodiesel production

**DOI:** 10.1186/2193-1801-2-475

**Published:** 2013-09-21

**Authors:** Efrén Aguilar-Garnica, Mario Paredes-Casillas, Tito E Herrera-Larrasilla, Felicia Rodríguez-Palomera, Daniel E Ramírez-Arreola

**Affiliations:** Departamento de Química, Universidad Autónoma de Guadalajara, Av. Patria 1201, Zapopan, Jalisco 44100 México; Departamento de Ingenierías, CU Costa Sur, Universidad de Guadalajara, Av. Independencia Nacional # 151, Autlán, Jalisco 48900 México

**Keywords:** Poly (styrene-co-butadiene), Sulfonation, Recycling, Catalyst, Biodiesel, Esterification

## Abstract

The disposal of solid waste is a serious problem worldwide that is made worse in developing countries due to inadequate planning and unsustainable solid waste management. In Mexico, only 2% of total urban solid waste is recycled. One non-recyclable material is poly (styrene-co-butadiene), which is commonly used in consumer products (like components of appliances and toys), in the automotive industry (in instrument panels) and in food services (e.g. hot and cold drinking cups and glasses). In this paper, a lab-scale strategy is proposed for recycling poly (styrene-co-butadiene) waste by sulfonation with fuming sulfuric acid. Tests of the sulfonation strategy were carried out at various reaction conditions. The results show that 75°C and 2.5 h are the operating conditions that maximize the sulfonation level expressed as number of acid sites. The modified resin is tested as a heterogeneous catalyst in the first step (known as esterification) of biodiesel production from a mixture containing tallow fat and canola oil with 59% of free fatty acids. The preliminary results show that esterification can reach 91% conversion in the presence of the sulfonated polymeric catalyst compared with 67% conversion when the reaction is performed without catalyst.

## Introduction

The Guadalajara Metropolitan Zone (GMZ) is the second largest urban area in México. More than 4 million inhabitants in the GMZ generate approximately 0.508 kgperson^-1^day^-1^ of household solid waste. The major components of the household solid waste are putrescible elements (53%), different types of paper (10%) and plastics (9%). From all of this waste, only 2.2% is separated for reuse/recycling, whereas the rest is deposited in municipal landfills. Rigid plastics, including poly (styrene-co-butadiene), represent approximately 1% of the non-recyclable materials (Bernache-Pérez et al. 2001).

On the other hand, it is well known that the widespread use of fossil fuel reserves has increased the air pollution levels worldwide, affecting global climate. These reserves (including petroleum-based diesel or petrodiesel) are being rapidly depleted. Biodiesel has been proposed as a renewable, biodegradable, non-toxic and non-inflammable alternative to petrodiesel. Chemically, biodiesel is a mixture of alkyl esters that is traditionally produced in a process known as transesterification in which refined plant oils or animal fats (i.e., triglycerides) are mixed with alcohol and heated in the presence of an alkaline catalyst. The relatively high cost of oils and fats contribute 60-80% of the total biodiesel cost, making it non-competitive with petrodiesel (Wen et al. 2010). To address the cost issue, it has been proposed that biodiesel be produced from cheaper feedstock, such as waste cooking oils (Liang 2013), grease from grease traps or animal fats (Canoira et al. 2008) that are characterized by their high (>1%) amount of free fatty acids (FFAs). However, the application of the alkaline transesterification technology to transform the aforementioned raw materials into biodiesel is not recommended because the reaction between FFAs and the alkaline catalyst makes soap, thereby reducing biodiesel conversion and creating difficulties in separating and purifying the product (Marchetti et al. 2007). To avoid this problem, it has been proposed that a pretreatment step be introduced before the conventional transesterification process. This pretreatment stage is known as esterification and is usually catalyzed by sulfuric acid, with reaction yields over 95% (Canacki and Van Gerpen 2001). Solid-acid catalysts, such as Dowex monosphere 550A, Dowex upcore Mono A-625, Amberlyst-15, Amberlyst-16, Amberlyst-35, Dowex HCR-W2, mesoporous aluminosilicates, Amberlyst 131, Relite CFS, ZrO_2_-supported metal oxide and mesoporous organosilicas, have also been considered (Özbay et al. 2008, Carmo et al. 2009, Tesser et al. 2010, Morales et al. 2010, Kim et al. 2011). More recently layered bismuth carboxylates, has also been used in esterification of fatty acids (Rosa da Silva et al. 2013). Compared with sulfuric acid, solid-acid catalysts have lower reaction rates, but they are often preferred over sulfuric acid because they are easily separated from the product, prevent corrosion (Silva and Rodrigues 2006) and can be reused (Vieira Grossi et al. 2010).

In this paper, we present a lab-scale strategy for recycling poly (styrene-co-butadiene) waste. Although the strategy is conceived to mitigate the solid-waste disposal problem in the GMZ, it could be extended to any city that has a similar situation. In this strategy, the poly (styrene-co-butadiene) waste is sulfonated with fuming sulfuric acid. The sulfonation method proposed here has been previously studied (Inagaki et al. 1999; Inagaki & Kiuchi 2001) but, to the best of our knowledge, the conditions (temperature and time) that maximize the number of acid sites in the sulfonation are not reported yet. Therefore, the behavior of the poly (styrene-co-butadiene) waste sulfonation under time and temperature variations is analyzed in the present work. This analysis is conducted with an experimental design from which is possible to deduce a mathematical model that adequately describes the sulfonation process. The theoretical optimal conditions for the sulfonation experimental runs are obtained from this model and the resulting product sulfonated in these conditions is then used as a solid-acid catalyst to produce biodiesel in the esterification of feedstock with a high content of FFAs.

## Materials and methods

### Materials

Poly (styrene-co-butadiene) waste was collected in the form of disposable cups. Chloroform, sulfuric acid and potassium hydroxide were provided by Analytyka (México). Methanol and phenolphtalein were obtained from Karal (México). Finally, fuming sulfuric acid and potassium bromide were provided by JT Baker (USA).

### Qualitative characterization of poly (styrene-co-butadiene) waste

Poly (styrene-co-butadiene) waste cups contain residual accumulations of carbonated drinks or natural/artificial juice. These cups are crushed to a particle size of approximately 0.60-0.80 cm^2^, washed in a detergent solution and then rinsed. The clean plastic pieces are dried to a constant weight and are qualitatively analyzed as follows to confirm the presence of butadiene. First, a 0.20 g aliquot of the polymer is dissolved in 2.5 mL of chloroform. The polymer is extracted from this solution with 10 mL of methanol to ensure that additives are not interfering with the characterization (Lacoste et al. 1996). The extract is dried and dissolved again in 2.5 mL of chloroform to generate two samples. A film obtained from the first sample is further analyzed with a Perkin-Elmer FT-IR spectrophotometer, whereas the second sample is added to bromine water.

### Sulfonation experiments

The sulfonation of poly (styrene-co-butadiene) waste which is already clean and dry, is carried out with fuming sulfuric acid (10 mL/g plastic) as the sulfonation agent. Different combinations of temperature (30°C, 70°C, 110°C) and time (1.0 h, 3.0 h, 5.0 h) are considered. Once the reactions are finished, the products are washed with distilled water and then dried to a constant weight.

### Sulfonation level determination

The sulfonation level of the sulfonated products is commonly known as number of acid sites and is expressed in terms of the number of milliequivalents of ~SO_3_H groups (*m*_*eq*_*SO*_3_*H*) per gram of sulfonated product. In this work, the sulfonation level was determined in a titration procedure with 0.1 N alkaline solution of potassium hydroxide using phenolphthalein as an indicator.

### Optimization of the sulfonation process

It is well known that the chemical reaction yield is strongly affected by temperature and time. Nevertheless the influence of these factors on the sulfonation process of poly (styrene-co-butadiene) is not reported yet. To cover this lack of information it is proposed in this work to conduct an experimental design considering three levels for each factor and the number of acid sites as response variable. The main objective of this 3×3 experimental design is to verify if a combination of temperature and time could maximize the number of acid sites. Previous experiments on the sulfonation process showed that a very low number of acid sites is obtained when the reaction is carried out at 30°C and 1.0 h. As a consequence, these operating conditions are set as the lowest point in the proposed design. Additionally, the highest point (i.e., 110°C and 5.0 h) is selected because it has been previously reported (Inagaki & Watanabe 2003; Inagaki and Noguchi 2003).

### Characterization of the product obtained under optimal sulfonation conditions

Poly (styrene-co-butadiene) waste is sulfonated at the optimal conditions that maximize the number of acid sites to both quantitatively and qualitatively characterize the sulfonated product and to verify whether it can act as catalyst in esterification reactions. The results of the quantitative characterization are expressed not only in terms of the number of acid sites but also in terms of methanol and water absorption, which are calculated following the “tea bag” method (Hosseinzadeh 2011). The qualitative characterization is carried out by using a sample to form a potassium bromide pellet whose infrared spectrum is recorded using a Perkin-Elmer FT-IR spectrophotometer (Martins et al. 2003).

### Esterification reactions

The polymer that is sulfonated under optimal conditions is tested as a catalyst in an esterification procedure. The raw material for this process is a synthetic mixture prepared by mixing tallow fat (supplied by Quimikao, Guadalajara, México) with commercial canola oil. Such feedstock is esterified with methanol in three different experiments: without catalyst, with sulfuric acid as catalyst (5% based on the FFAs weight in the mixture) and with sulfonated poly (styrene-co-butadiene) waste as catalyst. The main goal of these experiments is to explore the effect of the proposed catalyst on the conversion of FFAs for certain feedstock and to compare that effect with the effects generated both by the conventional catalyst and without catalyst. All the esterification reactions are carried out in batch conditions at 60°C and 1.0 h, with a methanol to FFAs molar ratio of 40:1 (Canacki and Van Gerpen 2001). At the end of the esterification, the reactants are allowed to settle in a separatory funnel. The conversion of FFAs (%*C*) in each reaction is computed with the following equation (Özbay et al. 2008; Carmo et al. 2009):1

where *A*_0_ is the initial content of FFAs for the feedstock and *A*_*f*_ is the final content of FFAs of the fluid extracted from the bottom of the separatory funnel (biodiesel phase). The values for *A*_0_ and *A*_*f*_ are obtained as described in the American Oil Chemists’ Society Official Method Cd 3d-63.

## Results and discussion

**Figure 1:**
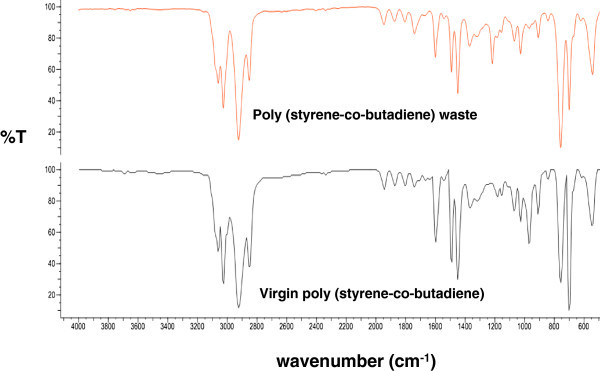
**Comparison between the spectrum of poly (styrene-co-butadiene) waste and the spectrum of virgin poly (styrene-co-butadiene).**

The infrared spectrum for the film from the clean and dry poly (styrene-co-butadiene) waste is taken over a range of wavenumbers from 600 to 4000 cm^-1^. This spectrum is compared with the corresponding spectrum of virgin poly (styrene-co-butadiene) (see Figure 1) by using the OMNIC E.S.P. software (Thermo Electron Scientific Instruments Corporation, Madison WI, USA). The match among these spectra is up to 80%.

Bromine water is added drop by drop to the chloroform-poly (styrene-co-butadiene) solution. The yellow-brown color of the bromine reagent disappears almost immediately when it contacts with the solution. This result confirms the presence of double bonds in the polymer (in addition to those related to the aromatic ring in polystyrene) and, therefore, the presence of butadiene. Thus, the results of the infrared test and the bromine water test proposed in subsection 2.2 suggest that the collected waste is indeed poly (styrene-co-butadiene). Once these tests are carried out, the polymer waste is sulfonated as described in subsection 2.3. The sulfonation level for each sulfonated polymer is computed as explained in subsection 2.4, with the results reported in Table 1.

**Table 1:** **Results of the sulfonation experiments expressed in terms of the number of milliequivalents of ~SO**_**3**_ **H groups per gram of sulfonated polymer**

Temperature time	30°C	70°C	110°C
**1 h**	0.42 ± 0.01	4.30 ± 0.17	2.66 ± 0.15
**3 h**	1.23 ± 0.06	5.13 ± 0.21	2.40 ± 0.20
**5 h**	0.82 ± 0.03	3.83 ± 0.12	**N**/**A**

It is important to note that a heterogeneous mixture of SO_3_ in sulfuric acid (i.e., fuming sulfuric acid or oleum) is used here as the sulfonating agent because it has a high reactivity due to the presence of SO_3_ (Kucera and Jancar 1998). As a consequence of the nature of this agent, one expects the presence of two phases (gas–liquid) while the sulfonation experiments are being conducted. This phenomenon is confirmed for all the experiments except for the one conducted at the operating conditions of 5.0 h and 110°C. In this case, two phases are observed at the beginning, but not at the end, where only the liquid phase remains; which most likely caused a reactivity decrease in the sulfonating agent. Therefore, to apply the same statistical treatment to all data, the operating conditions corresponding to 5.0 h and 110°C are considered to be unavailable in Table 1. The rest of the experimental data are analyzed with Statgraphics® (Centurion XVI, release 2009), which provides the following nonlinear regression model:2

In this equation, *N*.*A*.*S*. is the Number of Acid Sites of the polymer, *T* is temperature and *t* is time. Table 2 shows the results of an analysis of variance (ANOVA) carried out for testing the significance of each one of the regression coefficients in the model described by equation (2). If the level of significance (*α*) is chosen as 0.05 (i.e. 95% confidence level), the results in Table 2 demonstrate that all the coefficients are significant since p-value< *α*. Besides, it is also possible to conduct an ANOVA for testing the significance of the mathematical model. It is well known that the test procedure involves partitioning the total sum of squares (*S*_*ST*_) into a sum of squares due to the model (*S*_*SM*_) and a sum of squares due to the error (*S*_*M*_), say *S*_*ST*_ = *S*_*SM*_ + *S*_*E*_ where34

**Table 2:** **ANOVA for testing the significance of the individual regression coefficients**

Source	Mean square	p-Value
Temperature	3.2	0.0000
Time	0.95	0.0000
Temperature^2^	42	0.0000
(Time)(Temperature)	1.7	0.0000
Time^2^	2.8	0.0000

**Figure 2:**
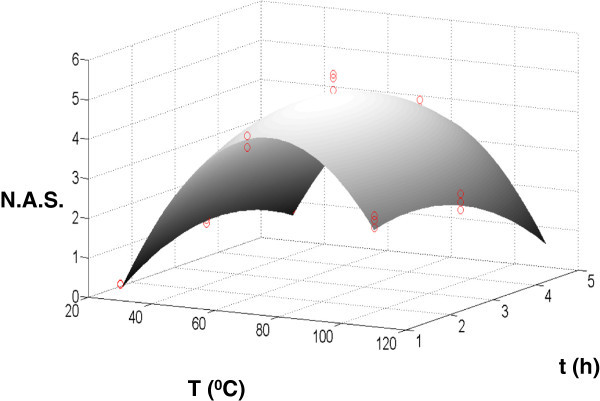
**Surface response for the proposed model in the sulfonation process.** Experimental data are plotted as red circles.

In equations (3) and (4), *n* is the total number of experimental data (observations), *y*_*i*_ denote the value of the experimental data (in this case the N.A.S.) at each temperature and time whereas  is the estimated value for the observations at each temperature and time obtained from the proposed model. From the sum of squares it is possible to compute the following statistic:  where *k* is the number of regressor variables in the model. If *F*_*O*_ exceeds *F*_*α*,*k*,*n*−*k*−1_ then the proposed model is significant at the level of significance *α* (Myers et al. 2009). The results for testing the significance of the model are shown in Table 3. If we select again *α* = 0.05 then *F*_0.05,5,18_ = 2.77. Since *F*_*O*_ > *F*_*α*,*k*,*n*−*k*−1_ then the model is significant at 95% confidence. From the results depicted in Table 3 is possible to compute the coefficient of multiple determination *R*^2^ and the adjusted statistic according with the following expressions:  and . For the proposed model, *R*^2^ = 0.982 and . This means that the proposed model explains in a satisfactory manner the variability observed. This fact is enhanced when the experimental results are plotted in the same figure than the proposed model (see Figure 2).

**Table 3:** **ANOVA for testing the significance of the proposed mathematical model**

Source of variation	Sum of squares	Degrees of freedom	***F***_***O***_
Model	61.2	5	202.397
Error	1.1	18	
Total	62.3	23	

**Figure 3:**
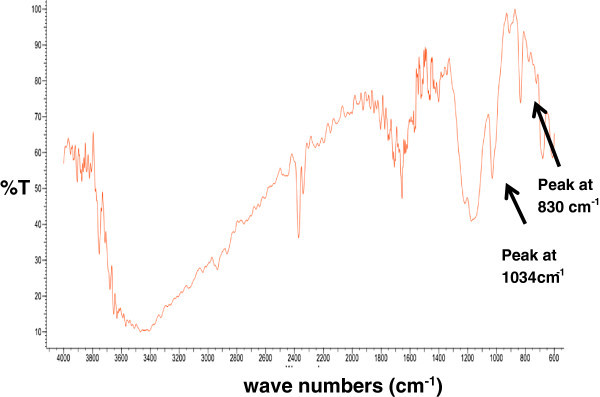
**Infrared spectrum of the sulfonated polymer prepared at optimal conditions.**

Once the model has been satisfactorily tested through diverse statistical tests, is possible to derive from such model the theoretical conditions that generate the maximum number of acid sites. These conditions are provided by Statgraphics and they correspond to *T* = 75°C and *t* = 2.5 *h*. Then, as described in subsection 2.6, the sulfonation reaction is carried out at these optimal conditions, and the resulting product is quantitatively characterized in terms of the number of acid sites , the methanol absorption capacity (1.7 g/g) and the water absorption capacity (7.2 g/g). This product is also qualitatively characterized with an infrared spectrum recorded over a range of wavenumbers, from 600 to 4000 cm^-1^ (see Figure 3). The peak at 830 cm^-1^ suggests the bonding of the ~SO_3_H groups to the aromatic ring of polystyrene. In addition, the absorption at 1034 cm^-1^ is a result of the symmetric stretching vibration of ~SO_3_H groups, (Martins et al. 2003). Once characterized, the polymer sulfonated under optimal conditions is tested as a catalyst for the esterification of a synthetic mixture with canola oil and tallow fat with 59% of FFAs (i.e. *A*_0_ = 59). This reaction is carried out with 0.12 g catalyst per gram of FFAs under the operating conditions depicted in section 2.7. The average conversion is computed with equation (1) and the result is 91%, with an easy recovery of the catalyst from the reaction products. In addition, the esterification reactions carried out with sulfuric acid and without catalyst achieve average yields of 99% and 67%, respectively. Both yields were also computed considering equation (1). Finally, it is important to state that the objective of this paper is not to make an extensive characterization (e.g. thermal stability analysis, morphological charac-terization) of the product sulfonated under optimal conditions. The goal of the paper is to analyze the sulfonation level for poly (styrene-co-butadiene) waste under time and temperature conditions and also to verify the catalytic activity of sulfonated polymer in optimal conditions. The qualitative characterization of the aforementioned polymer based on its IR-spectrum was presented here to demonstrate that it contains ~SO_3_H groups. Probably, these groups are responsible for the satisfactory activity of the proposed catalyst since they are considered as active sites in the Eley-Rideal mechanism which is assumed to occur in esterification reactions promoted by solid-acid catalysts (Tesser et al. 2010).

## Conclusions and perspectives

Poly (styrene-co-butadiene) waste was sulfonated with fuming sulfuric acid under varying times and temperatures. The objective of these experimental runs was to apply a 3×3 experimental design to deduce a mathematical model that adequately represents the sulfonation data at 95% confidence. From this model is possible to derive the operating conditions (75°C and 2.5 h) maximizing the number of acid sites in the sulfonated polymer. Then, it was verified that the sulfonated polymer waste under these conditions was able to act as catalyst in the esterification of a synthetic mixture of tallow fat and canola oil with a high FFAs content. Thus, it was demonstrated that this type of rigid plastic waste can be treated and further applied in a process that produces biodiesel. However, there is another issue related to the proposed catalyst that remains to be examined: its stability (i.e., the catalytic activity when the catalyst is reused). We are currently studying this issue, along with the economic feasibility of the proposed catalyst which is crucial to scale up the strategy proposed here from lab-scale to pilot-plant scale. Another future research topic is a comparison between the activity of the proposed catalyst and the activity of other catalysts that are either reported in the literature or commercially available.

### Nomenclature

*GMZ* Guadalajara Metropolitan Zone.

*FFAs* Free Fatty Acids.

*A*_*O*,_*A*_*f*_ Initial and final content of FFAs in the esterification experiments, respectively.

*N*.*A*.*S* Number of Acid Sites.

*T*,*t* Temperature and time, respectively.

*S*_*ST*_,*S*_*SM*_,*S*_*E*_ Total sum of squares, sum of squares due to the model and sum of squares due to error, respectively.

*y*_*i*_, *ŷ*_*i*_ Experimental data and estimated value, respectively.

*F*_0_ Stastistic and significance level, respectively.
